# Intensify3D: Normalizing signal intensity in large heterogenic image stacks

**DOI:** 10.1038/s41598-018-22489-1

**Published:** 2018-03-09

**Authors:** Nadav Yayon, Amir Dudai, Nora Vrieler, Oren Amsalem, Michael London, Hermona Soreq

**Affiliations:** 1 Department of Biological Chemistry, The Life Sciences Institute, The Hebrew University of Jerusalem, Jerusalem, Israel; 2 The Edmond and Lily Safra Center for Brain Sciences (ELSC), The Hebrew University of Jerusalem, Jerusalem, Israel; 3 Department of Neurobiology, The Life Sciences Institute, The Hebrew University of Jerusalem, Jerusalem, 9190401 Israel

## Abstract

Three-dimensional structures in biological systems are routinely evaluated using large image stacks acquired from fluorescence microscopy; however, analysis of such data is muddled by variability in the signal across and between samples. Here, we present Intensify3D: a user-guided normalization algorithm tailored for overcoming common heterogeneities in large image stacks. We demonstrate the use of Intensify3D for analyzing cholinergic interneurons of adult murine brains in 2-Photon and Light-Sheet fluorescence microscopy, as well as of mammary gland and heart tissues. Beyond enhancement in 3D visualization in all samples tested, in 2-Photon *in vivo* images, this tool corrected errors in feature extraction of cortical interneurons; and in Light-Sheet microscopy, it enabled identification of individual cortical barrel fields and quantification of somata in cleared adult brains. Furthermore, Intensify3D enhanced the ability to separate signal from noise. Overall, the universal applicability of our method can facilitate detection and quantification of 3D structures and may add value to a wide range of imaging experiments.

## Introduction

**Figure 1:**
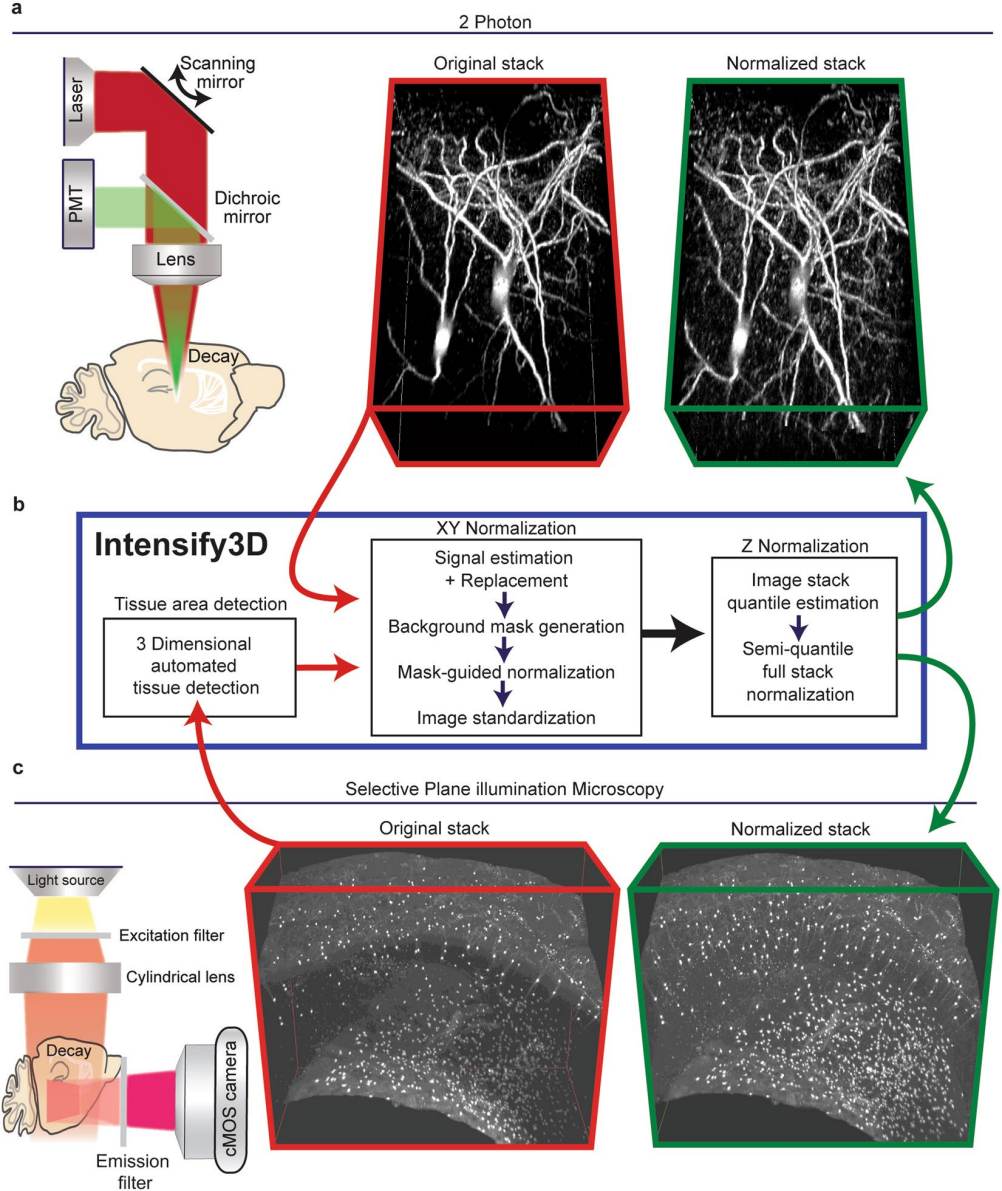
The basic normalization process of Intensify3D for 2-Photon and Light-Sheet 3D imaging (**a**). Left panel. 2-photon imaging setup illustrating the decay in excitation laser (red) and emitted light (green) through the imaged tissue. Red frame, middle panel. 3D projection of *In vivo* 2-photon Z-stack of CChIs up to 300 µm depth, bottom portion is deeper. Green frame, right panel. 3D projection of image stack post normalization with Intensify3D; note the enhanced visibility of deep neurites (**b**). Intensify 3D processing pipeline for 2-Photon and light sheet image stacks. The latter requires an additional step to only account for tissue pixels in the image. The images in the stack are normalized one by one (XY normalization). After all the images are corrected the entire stack is corrected (Z Normalization) by semi-quantile normalization (other options exist) (**c**). Left panel. Light-Sheet imaging setup where the excitation light is orthogonal to the imaged surface. Red frame, middle panel. iDISCO immunostaining and clearing of CChIs as well as striatal Cholinergic interneurons. Original image suffers from fluorescence decay at increasing tissue depth. Green frame, right panel. Intensify3D Normalized image stack. Images before and after normalization are presented at the same brightness and contrast levels.

Fluorescence microscopy once relied on single plane images from relatively small areas, and yielded limited amounts of quantitative data^1^. Nowadays, many imaging experiments encompass some form of depth or a Z-stack of images, often from distinct regions in the sample. Hence, much like biochemical and molecular experimental datasets^2,3^, accurate normalization, beyond background subtraction^4^ of imaging signals, could reduce tissue-derived and/or technical variation. Signal heterogeneity often arises from sample-specific factors (e.g. excessive blood vessel absorbance in live imaging, or non-uniform tissue clearing/antibody penetration in fixed tissues). These elements combined with imaging distortions and illumination gradients contribute to non-uniformity both within and across image stacks and may lead to erroneous conclusions. Such heterogeneity is exacerbated the larger the imaged structure and it often limits the ability to perform downstream applications such as feature extraction, threshold-based detection, co-localization, three dimensional (3D) rendering, and image stitching. Standard filtering as well as total image correction tools that construct a mathematical model based on multiple single plane images^5–7^ may excel at improving specific types of shading or microscopy distortions. However, they do not account for differences that arise from sample specific factors and are sub-optimal when signal-to-noise ratios, imaging conditions, and pixel distributions vary in a location-dependent manner – a typical property of 3D imaging. Specialized image processing tools for brain datasets have been designed to correct signal homogeneity but are limited to a specific use (e.g. somata detection)^8,9^. Moreover, modern 3D image datasets are acquired using advanced imaging modalities^10–12^ and are based on novel sample preparation techniques^13–18^, some leaning on open source analysis tools^19,20^. Specifically, 2-Photon (2P) and Light-Sheet (LS) microscopes enable the acquisition of images from both deep and wide tissue dimensions (Fig. 1a,c, left panel). However, every biological sample and imaging technique introduces its own acquisition aberrations: beyond mirror and lens distortions^21^, the imaged preparations combine different characteristics (of e.g. cell density and lipid composition) that affect the optical penetration and light scattering at diverse tissue depths. Experimental limitation (antibody penetration, clearing efficiency) also constrain the ability to extract information from imaging experiments. Taken together, these difficulties call for the development of universal post-acquisition image correction/normalization tools that account for signal-carrying pixels and which estimate the specific heterogeneity of each image individually.

To achieve 3D normalization, we developed a new algorithm, Intensify3D (Fig. 1b), based on the following basic assumptions: (1) In the “perfect imaging experiment”, the intensity distribution of the background would have been similar throughout the imaged region; to achieve this quality, differences in background intensity distribution should be corrected by Intensify3D. (2) In many microscopy images, the fraction of signal-carrying pixels is significantly smaller than that of the background pixels. Moreover, signal-portraying pixels are often sparse and variable across the imaged region, while some images in an image stack might not contain a signal at all. On the other hand, background pixels are (by the assumption above) numerous and exhibit a continuous pixels histogram (often following a Poisson^22^ distribution), allowing accurate assessment of quantiles. Leaning on these features, Intensify3D aims to detect and use the background for correct normalization of the signal. Consequently, our normalization algorithm initiates with an estimation of the background by removing as much as possible of the imaged signals. Then, the background intensity gradients are used for correction by local transformation (correction by division) of both signal and background, without compromising the signal-to-noise ratio (Fig. 1a,b).

## Intensify3D stack normalization: methodological outline

We selected 2 P *in vivo* brain images harboring fluorescently labeled Cortical Cholinergic interneurons^23^ (CChIs), which present with challenging complexity and diversity, to demonstrate our capacity to reach enhanced signal uniformity across the entire 3D space. Our correction process employs two input parameters that are determined by the user and represent the imaged signal: (1) Maximum background intensity (MBI), which stands for the highest pixel value of the background in a selected image stack. (2) Spatial filter size (SFS), which should be determined based on the largest element in the signal and preferably be at least twice the size of a typical imaged structure (Supplementary Figs S1 and S2a). Based on the MBI, Intensify3D automatically assigns a matching value to the entire image stack (Supplementary Fig. S2b). Initially, each image in the stack is normalized separately across the XY dimensions. To generate an accurate representation of the image background, the signal carrying pixels are deleted by applying a threshold (MBI) and replaced by values presenting similar distributions to that observed in the rest of the image (Supplementary Fig. S2c). Next, a background mask image is created by a Savitzky-Golay spatial filter (SFS), further removing features of the signal from the mask image while preserving general intensity gradients in the background (Supplementary Fig. S2d, middle panel). Note that larger values of SFS will result in normalization of larger scale gradients in background intensity while ignoring smaller spatial changes. After the mask image is generated, it is used for normalization by division: the value of each pixel I(x) in the original image, I, is divided by the value of the corresponding pixel in the mask image, M, to produce a corrected image, N (*For every pixel x*, *N*(*x*) = *I*(*x*)/*M*(*x*)) (Supplementary Fig. S2d). The corrected image is then standardized to avoid artificial “overexposure” due to normalization. Finally, for normalization across the imaged stack, Intensify 3D offers 3 types of Z normalization: (1) Upper quantile normalization, which shifts the intensity histogram of each image so that the upper quantile (based on MBI) would match across the entire stack (Supplementary Fig. S3a). (2) Contrast stretch normalization, which fits the intensity histogram to two intensity quantiles (tenth percentile and upper quantile) through linear interpolation (Supplementary Fig. S3b). (3) Semi-quantile normalization, which matches all image quantiles up to the upper quantile across the stack. Based on the transformation of the quantiles, pixels higher than the upper quantile are corrected through contrast stretch (Supplementary Fig. S3c). Semi-quantile normalization achieved the best results in terms of homogeneity of both background and signal throughout the stack. (Figure 1a,c, Green frames).

## Addressing background complexity

For cases where the imaged sample does not occupy the entire image (Fig. 1c), Intensify3D includes an option to automatically detect the area of the tissue (Supplementary Fig. S4) and thus avoid normalization of irrelevant areas (e.g. imaging media) of the image. This feature is especially important when the relative size of the tissue section changes dramatically across the stack, as is often the case with Selective Plane Illumination Microscopy (SPIM) of large tissue samples (e.g. brain, heart). The automated detection option is based on principal component analysis (PCA) followed by either the application of a Gaussian mixture Expectation Maximization (E.M.) algorithm or K-means clustering to detect pixels that belong to the tissue. This step minimizes possible normalization artifacts due to media/tissue borders across the stack and accounts for changes in tissue size across the stack.

Supplementary Fig. S1 presents a MATLAB graphical user interface (GUI) manual for using Intensify3D.

## Results

To challenge the value of the Intensify3D normalization algorithm, we used in-house data from 2P and LS brain, mammary gland, and heart image stacks as well as simulated data. These represent distinct types of modern imaging platforms that are used for both visualization and quantification analyses and produce vast 3D data that can gain substantial additional value when normalized. Notably, each of these techniques introduces its own constraints both at the step of sample preparation and during the imaging process (detailed below); and our tool comes to correct both of these aspects.

### Correction of *in vivo* 2-Photon imaging data facilitates accurate neurite detection and measurements

In a typical 2P brain imaging experiment, a cranial window is opened in the mouse skull and the gap between the objective lens and the surface of the brain is filled with a water-based medium (external buffer or gel). 2P excitation is achieved through a tunable near infra-red pulsed laser, and the emitted fluorescence is split by filtering the image through green and red light filters, yielding split signals that are detected by photomultiplier tubes (PMTs)^24^. However, both the excitation light and the emitted light are subject to depth-dependent scattering; therefore, the excitation gradually becomes less efficient, which limits the power of detection with increasing depth despite the same amount of power being used. In addition, the detected photon emission is diminished accordingly, which leads to signal decreases both in intensity and in resolution (Fig. 1a, left panel). Another source of inhomogeneity comes from the different reflective indices of diverse biological materials; for example, blood vessels absorb red light more than the surrounding tissue^24^. Thus, both the tissue and the imaging technology cause distinct difficulties, each of which needs correction to achieve appropriate normalization.

**Figure 2:**
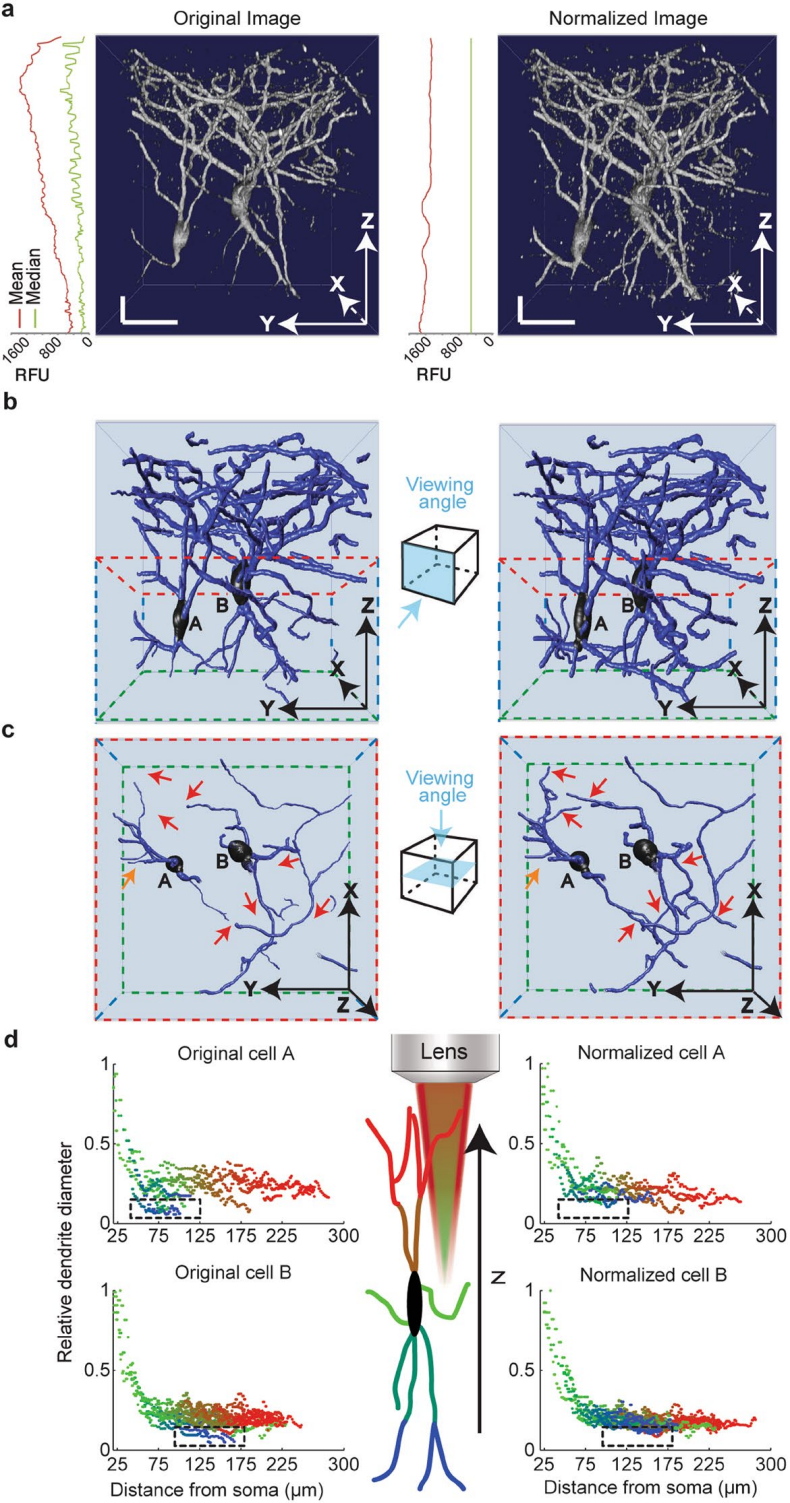
Intensify3D normalization of 2-Photon imaging data corrects and facilitates the reconstruction of CChIs (**a**). Orthogonal projections of 3D rendered image stacks before (left) and after (right) normalization. Images are shown together with mean (red) and median (green) relative fluorescent units values across all imaging planes. Note the homogeneous average and median intensity levels post-normalization. Horizontal and vertical scale bars represent 25 µm. For presentation purposes, the z dimension is smaller than x and y dimensions (**b**). Semi-automated reconstructions of the 2 CChIs (**A** and **B**) image stacks above, before and after normalization. Side boxes present the directions of view (**c**). Top view of the deeper portion (red and green frame) of reconstructed CChIs. Red arrows mark deep dendrites that were detected in the normalized, but not the pre-normalized reconstruction; orange arrow represents the reciprocal dendrites (**d**). Comparison of the diameter of deep to superficial (blue to red gradient represents Z depth) neurites as a function of distance from the soma. This analysis is based on the reconstructions of both CChIs. Notice that pre-normalization deep dendrites (black rectangles) seem smaller in diameter compared to superficial ones of similar somatic distance. In comparison, post-normalization dendrites show similar diameters to superficial dendrites of similar somatic distance. Illustration of lens and red excitation light illustrates the direction of illumination and decay of light as a function of depth.

The membrane composition, dendritic and axonal dimensions, and the morphology of neurons together determine their function^25–27^, making accurate assessment of a neuron’s structure crucial to understanding the scope of its performance. Cortical cholinergic interneurons (CChIs)^23^ provide an intriguing example of a neuronal population with functional complexity^28^. To access this specific neuronal population, we used mice that endogenously express a red fluorescent protein (ChAT_Cre X loxp_stop_loxp_tdTomato) in all cholinergic neurons. We then acquired 2P image stacks through a cranial window in an anesthetized mouse, with the same laser intensity across all depths (30 to 300 µm) (see *Methods*). Applying the Intensify3D normalization algorithm on this image stack added ample details to the observed structures without compromising their basic features. This is demonstrated by homogeneous image statistics, represented by the median and mean values across stack depths (Fig. 2a and Movie 1). To estimate the difference between pre- and post-normalization images for feature extraction capabilities, we reconstructed neurons from original and corrected image stacks by a “blind” experimenter using a semi-automated reconstruction tool (Vaa3D, Allen Institute)^29^ (Fig. 2b). This reconstruction highlighted considerable increases in the numbers and complexities of deep neurites (Fig. 2c). It further presented superior uniformity of dendritic diameters (automatically assigned by the reconstruction software) between deep and superficial dendrites (Fig. 2d), compatible with the known features of this class of bipolar cortical interneurons^23^. The apparent depth-dependent variability of dendritic diameters in the original 2P Z-stack is therefore misleading, and Intensify3D corrects this erroneous depth dependent profile of the normalized stack which is consistent with the actual situation^30^ (Supplementary Fig. S5). Our algorithm thus expanded the capacity to detect and reconstruct deep neurites while maintaining their spatial characteristics and correcting 3D microscopy errors.

### Normalized Light Sheet microscopy images enable precise identification of anatomical macro- and microstructures

Aside from the difficulties of imaging deep details of cellular features, normalizing microscopy image stacks is often confronted with large scale imaging variability. We addressed this issue using Light Sheet (LS), or Selective Plane Illumination Microscopy^31^ (SPIM). LS microscopy differs fundamentally from confocal and 2P imaging in that the excitation involves a single sheet-like beam that is projected orthogonally to the acquisition objective, and in that the image is captured by a CMOS camera instead of the scanning laser in 2P^12^. This offers a powerful capacity for preparing multiple micrographs from vast areas of transparent tissue samples in a short time, while avoiding damage to tissue preparations. However, this technology also involves a major challenge in achieving equal penetration efficacy of the light beam through the specimen as well as of antibody penetration if used in combination with immunostaining. Reflections, deflections, and diffractions caused by differences in the intrinsic characteristics of the tissue (e.g. white vs. grey brain matter, cavities, etc.) as well as from the angle at which the light enters the tissue may additionally distort the signal in a plane-specific manner and result in non-homogeneous excitation.

### Extraction of accurate barrel field anatomy from auto-fluorescent LS scans

**Figure 3:**
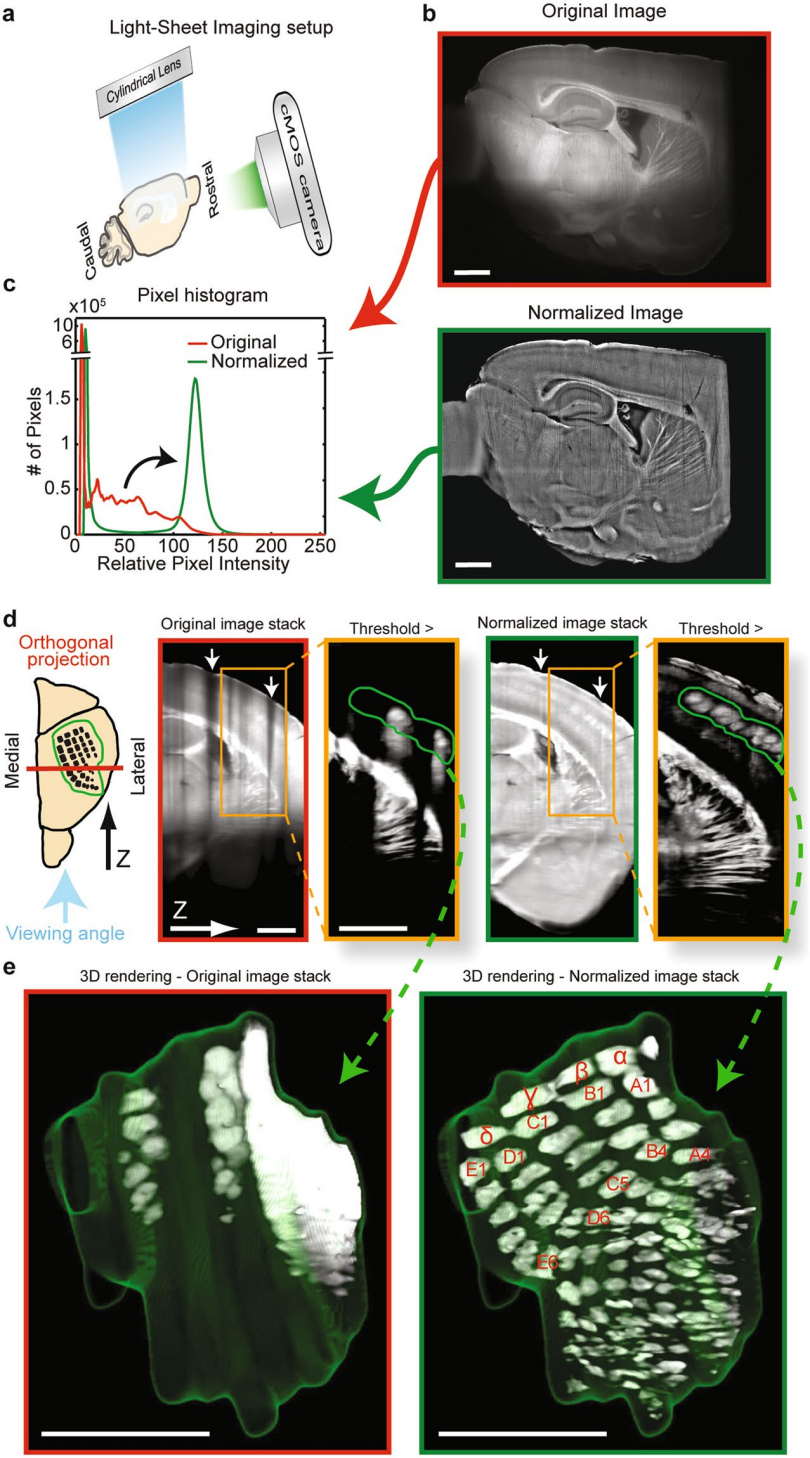
Auto-fluorescence Light-Sheet image normalization reinforces the iDISCO 3D detection of barrel fields (**a**). Imaging setup of cleared brain samples with a LS microscope. The LS blue excitation illumination plane is perpendicular to the filtered CMOS camera (**b**). A single representative sagittal scan. Shown is the blue/green excitation emission spectrum of cleared mouse hemisphere samples before (red frame) and after normalization (green frame) (**c**). Relative (matched minimum/maximum values) pixel intensity histograms of images before and after correction. Note the post-normalization shift of pixels (black curved arrow) that corresponds to tissue and not to background pixels (**d**). Pre- and post-normalized image stacks perpendicular to imaging plane, see orientation illustration. Pre-normalization image stack shows decreased intensity due to grooves in the tissue surface (white arrows) as well as along the path of illumination (down). Orange rectangle region emphasizes the barrel cortex region. After applying a threshold (pixels below threshold removed), a 3D region around the barrels was selected (green region) for 3D rendering (FIJI) (**e**). 3D rendering of barrel fields before and after image normalization. Annotation for barrels marked in red, the green mesh labels the region of interest. Scale bars are 1 mm.

To test the capacity of Intensify3D to overcome difficulties at the macro scale level, we selected the cortical barrel fields which may be visualized in the auto-fluorescent channel of cleared hemi-brain iDISCO preparations^20^. Barrel fields present an intriguing example of a spatially defined, cortical processing unit capable of experience-dependent rewiring^32^. Recent studies have shown the importance of precise mapping of neuronal types in a single barrel column^33^ and the effect of this anatomical diversity on network activity patterns^34^. Thus, the identification of individual barrel fields is crucial for studies focused on this region. Figure 3 presents an LS scan in the auto-fluorescent blue/green excitation emission spectrum of cleared mouse hemisphere samples prepared with the iDISCO+ method. Such scans may provide ample information regarding diverse neuroanatomical macrostructures^13^ (e.g. white and grey matter, barrel cortex composition, hippocampus areas, blood vessels, etc.) without external fluorescent labeling. However, this type of signal is inclined to photo bleaching and suffers from massive changes in intensity along the path of the LS beam through the tissue (Fig. 3a,b top panel). This poses a challenge when attempting to select a threshold to separately identify elements within the tissue or between the tissue and the imaging media.

At the single image level, Intensify3D corrected for intensity differences in the XY dimensions (Fig. 3b). Such corrections resulted in a shift in intensity of pixels (Fig. 3c, black curved arrow) of the tissue but not the media background due to automated tissue detection (Supplementary Fig. S4). In post-normalization images, a simple threshold could then differentiate between the distinct anatomical features within the tissue (Fig. 3c, Movie 2). However, in addition to the X and Y dimensions, the original image stack showed substantial differences in intensity between different scans along the Z-axis. In our example, this was probably due to grooves in the surface of the tissue (Fig. 3d, arrows, Movie 2), which became more apparent after applying a threshold in an attempt to separate between distinct barrel structures (Fig. 3d, orange box - green region). Consequently, our correction contributed to improved homogeneity also along the Z-axis, allowing the selection of a single threshold by which each of the barrel structures could be effectively separated from the background around them. After 3D rendering (ImageJ, 3D viewer)^35^, all of the principal barrels^36^ were clearly identified and could be numbered (Fig. 3e, Movie 3), further offering the option of testing and comparing their structural features individually for comparative analyses of different experimental samples.

### Correction of antibody and light penetration with Intensify3D facilitates accurate soma detection and quantification

**Figure 4:**
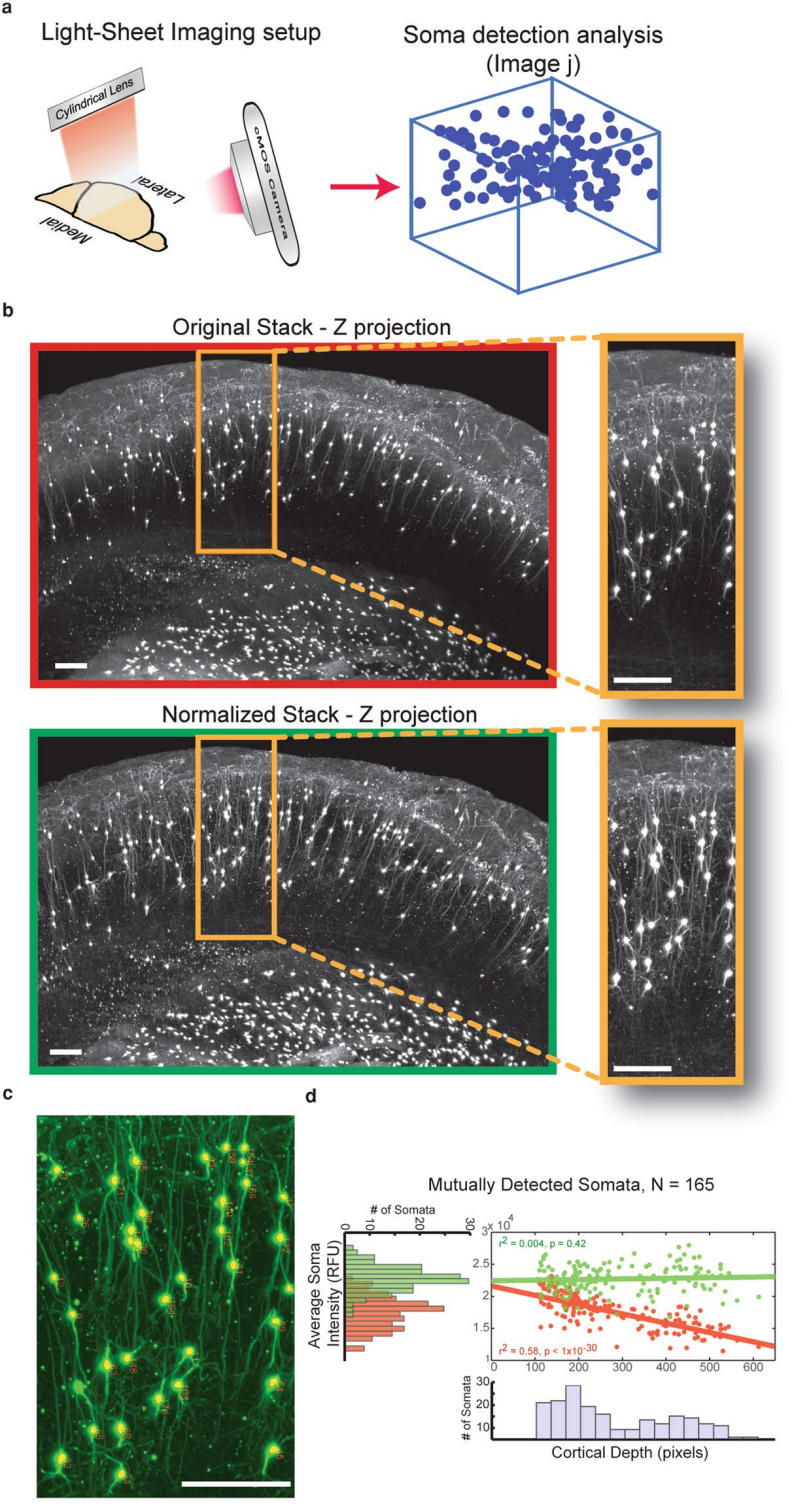
Intensify3D enables accurate detection and quantification of CChIs in deep cortical layers (**a**). Imaging setup of LS microscope. A z stack 1mm deep was acquired by far red excitation/emission LS scan of a iDISCO full cortical perpetration (**b**). Z projection of 250 images showing the CChIs as well as striatal cholinergic interneurons. Orange frame highlights the CChIs. Note the decay in background fluorescence with tissue depth in original image stack enhanced visibility and homogeneity of deep dendrites of CChIs in corrected image stack (green frame) (**c**). Representative enlarged region overlaid by results of soma detection analysis (yellow dots and red numbers) (**d**). Soma detection analysis (Fiji, See Methods). Mutually detected somata (from original and corrected image stacks) show a decrease in intensity (Pearson correlation R = −0.76, P < 1 × 10^−30^) and a wide distribution (red histogram). The somata intensity in corrected image stacks shows no correlation with cortical depth ((Pearson correlation R = 0.063, P = 0.42) and a narrower distribution (green histogram). All scale bars are 50 µm.

The power of the LS microscope effectively comes into play when combined with tissue clearing techniques. The ability to acquire microscale morphologies and cellular distributions in a preserved macroscale tissue within a short time is unique to this technique. The iDISCO technique offers superb clearing power and the ability to immuno-stain desired targets and use far-red fluorophores that are superior in terms of interference by auto-fluorescence (Fig. 4a). Nevertheless, variabilities in LS laser efficiency and antibody penetration efficacy both contribute to heterogeneities in the signal (Fig. 1c, left panel). Thus, cholinergic interneurons that are sparsely distributed within cortical layer 2/3 are easily visualized in this technique, but assessing their numbers, locations, and morphologies within cleared brain samples is confronted with in-depth limitations (Fig. 4b, top panel).

To test the capacity of Intensify3D to correct LS images for microstructure analysis we used the iDISCO method on cortical tissues of mice where all cholinergic neurons express a red fluorophore and stained these cells with a far-red dye. We imaged a 0.8 × 1 mm area of the cortex and applied Intensify3D with automated tissue detection. Post-normalization images showed superior uniformity of imaged neurons, enhancing neuronal morphologies (Fig. 4b, bottom panel). Finally, we applied an open-source analysis tool (Fiji, 3D object counter)^37^ to detect the somata of the CChIs and measured the distance of each soma to the cortical surface. Detected somata from original image stacks showed declining soma intensities as a function of cortical depth, most likely as an effect of decreased penetration of light and/or staining antibody. This reduction has been corrected with images normalized by Intensify3D (Fig. 4c,d). Specifically, the somata intensities in corrected image stacks showed no correlation with cortical depth (Pearson correlation R = 0.063, P = 0.42.) and a narrower distribution (Fig. 4d). Our analysis tool thus enabled correct assessment of both the site and density of these neuronal populations at variable tissue depths.

### Intensify3D restores distorted artificial 3D data and facilitates quantification of detected spheres

**Figure 5:**
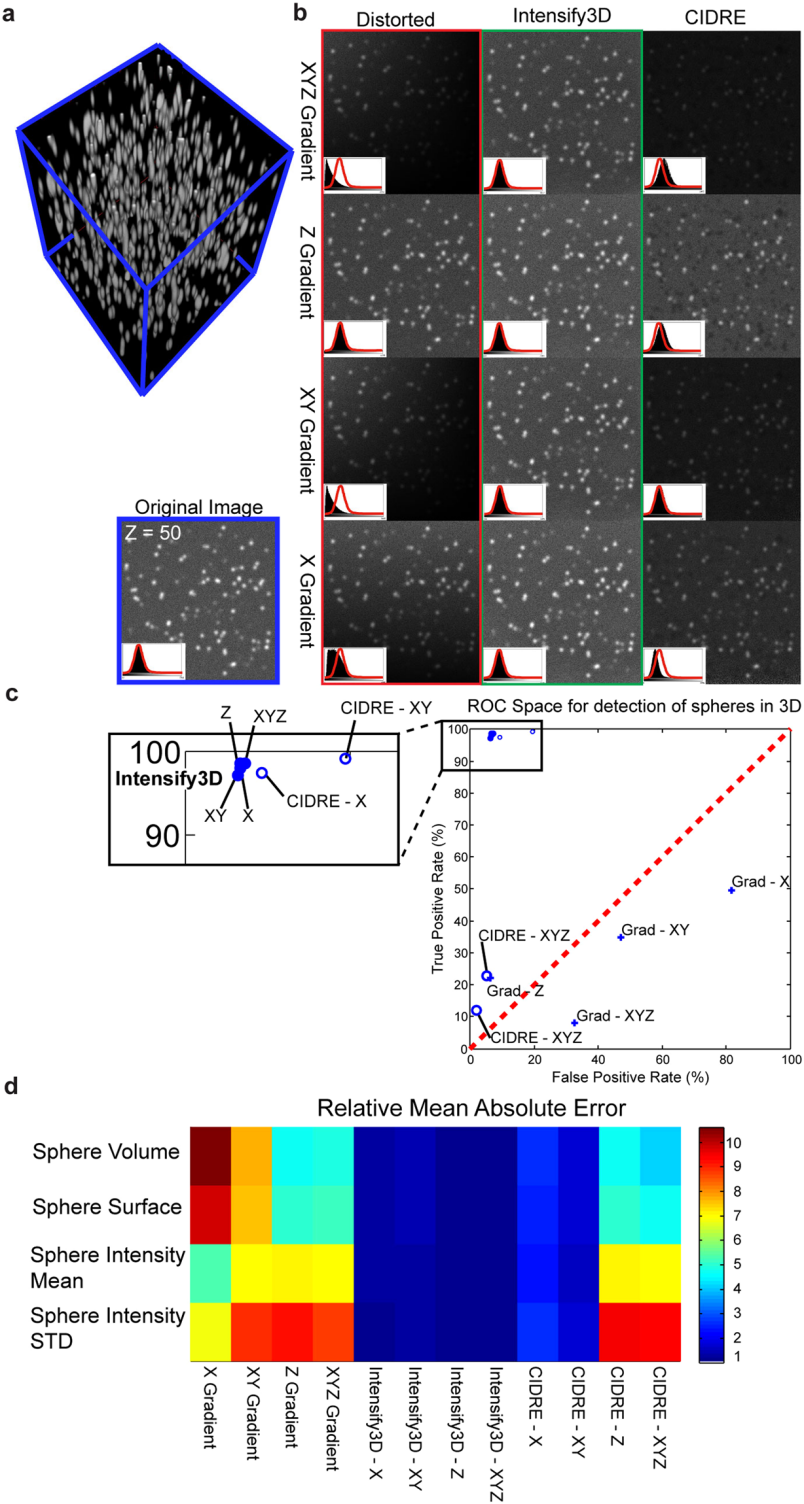
Intensify3D restores distorted artificial 3D data and facilitates quantification (**a**). 3D rendering of undistorted artificial 3D image stack of ~500 Gaussian spheres. Image stack dimensions – X Y Z: 600 × 600 × 500 pixels (**b**). Representative image (Z = 50) of undistorted image stack (left bottom) and distorted image stacks before and after correction with Intensify3D or CIDRE. All images are presented at the same minimum/maximum brightness levels. Outline of intensity histogram for the undistorted image (red curve) is overlaid on top of black filled intensity histograms for each of the individual images (left bottom corner) (**c**). ROC space for true/false positive detection rates by 3D object counter plugin (FIJI) on distorted data (crosses), Intensify3D (filled circles) or CIDRE (empty circles) corrected. Performance is in comparison to undistorted data (**d**). Relative mean absolute error for measured statistics of true positive spheres for distorted data, Intensify3D or CIDRE corrected. Performance is in comparison to undistorted data. Each row was corrected so that the minimal error is 1.

To supply controlled estimates of the performance of intensify3D we created an artificial image of randomly scattered 3D spheres. The artificial data is composed of ~500 Gaussian spheres with an artificial point spread function and an added background and Gaussian noise (Fig. 5a). We then applied the following intensity gradients to the 3D image: (1) Linear along the X axis. (2) Linear along both X and Y. (3) Logarithmic along the Z axis, and finally (4) Combined linear along X and Y together with a logarithmic gradient along the depth axis-Z. We corrected each distortion with either Intensify3D or CIDRE^6^ (Fig. 5b). Intensify3D managed to restore the shape and pixel proportion in all cases without showing any visible artifacts in the corrected data (Fig. 5b, red histograms). In comparison, images corrected with CIDRE displayed “black spots” in the background, probably due to interference from the signal. Finally, we estimated the correction by applying the 3D object counter function (FIJI)^19^ to detect and measure the spheres compared to the original undistorted data (Fig. 5b, Blue frame). Intensify3D performed better than CIDRE in all cases. Predictably, CIDRE did not account for changes in depth (Z gradients) since it is not designed for 3D analysis (Fig. 5c). Also, we selected the true positive spheres from both uncorrected images or those corrected by Intensify3D or CIDRE, and estimated the difference between original and corrected data (Mean absolute error). Again, correction with Intensify3D produced the lowest scores in all conditions (Fig. 5d).

### Intensify3D is applicable for a large range of biological tissues

**Figure 6:**
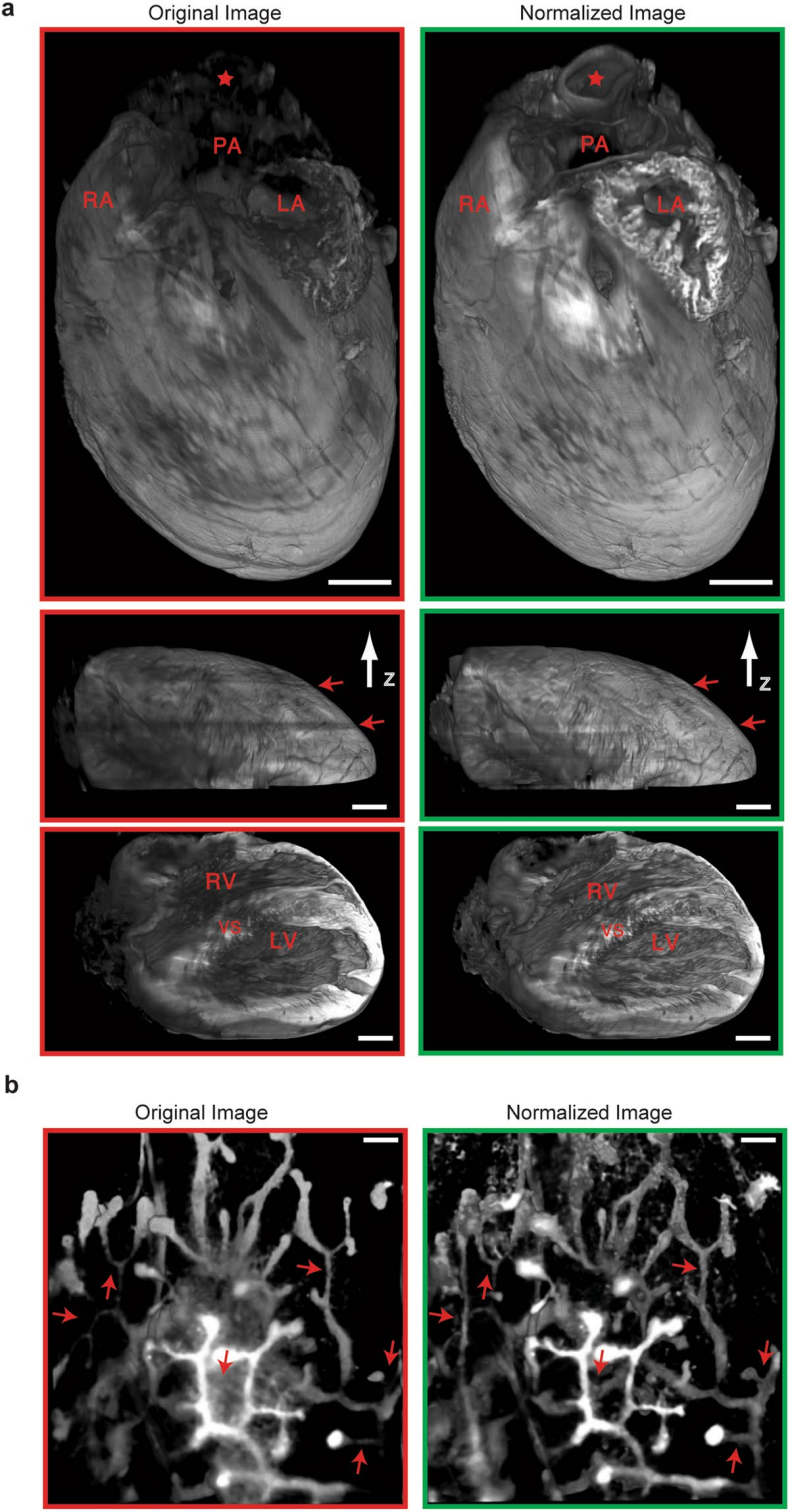
Correction of SPIM Imaging of mammary gland ducts and heart anatomy (**a**). Correction of a cleared iDISCO heart with intensify3D. 3 views (top, middle and bottom panels) of 3D rendering (ImageJ) based on whole mount mouse heart before (red frames) and after (green frames) correction. Aorta (red star), pulmonary artery (PA), left atrium (LA), right atrium (RA), right ventricle (RV), left ventricle (LV) and ventricular septum (VS) are marked. Scale bar represents 1mm in all views (**b**). 3D rendering based on whole mount imaging of cleared mouse mammary gland ducts before (red frame) and after (green frame) normalization with Intensify3D. Scale bar presents 150 µm. Left and right panels are matched in brightness and contrast.

To test the ability of Intensify3D in normalizing a variety of biological imaging datasets we chose two well-described complex structures: (1) the mouse mammary milk ducts and terminal end buds^38^ and (2) the mouse heart^39^. Both samples were cleared with the iDISCO technique and imaged with a LS microscope in the auto-fluorescent channel as described above. The heart sample showed impressive uniformity across the imaged tissue, allowing classification of the major heart arteries and ventricles (Fig. 6a, upper panel). Notice the correction of dark frames along the imaging path (Fig. 6a, middle panel, red arrows). Likewise, mammary milk ducts post-correction presented enhanced features, enabling detection of distal ducts and buds with the same threshold (Fig. 6b).

## Discussion

When the neuroscience pioneers –Santiago Ramón y Cajal, Camillo Golgi, and Alois Alzheimer, to name a few – drew beautiful neuronal structures based on their basic microscopes, they likely overcame image inhomogeneity and imaging limitations with the help of a keen eye and much experience. Today, manual drawings and descriptive microscopy have been replaced by high resolution, large scale data which call for accurate quantification; moreover, signals that seem clear by eye do not always translate well to the downstream computerized tools. To address these difficulties, we developed and tested a post-imaging normalization tool in two state-of-the-art imaging platforms, and demonstrated that it can overcome common sample heterogeneity in large image stacks using both of these technologies and correct significant dataset errors. Specific advantages of our algorithm include its capacity to distinguish between the signal and background with minimal parameters defined by the experimenter, and avoiding distorting one at the expense of the other, as well as enabling applicability to various imaging platforms. The resulting avoidance of imaging errors and improvements in signal homogeneity are therefore an important asset for fluorescence microscopy imaging studies of all cells and tissues, especially in the brain.

### 2-photon imaging

Numerous microscopy studies require viewing large fields while maintaining high resolution and keeping the accuracy of microstructures. Furthermore, enabling accurate semi- or fully-automated reconstruction of microstructures from large image stacks is a prerequisite for a number of ambitious research efforts, including the Blue Brain project^40^ and the BigNeuron initiative^41^. In this context, we challenged the use of our Intensify3D tool by analyzing 2P microscopy image stacks of adult mouse brains with fluorescently labeled cortical cholinergic interneurons^23^. Intensify3D normalization enabled homogenous representation across the entire image stack. Additionally, Intensify3D corrected significant errors in the estimation of deep dendrite diameters. Thus, normalized images offer a better representation of both imaged cell bodies and their thin extensions and serve as a superior platform for reconstructions and possibly modeling of the electrical properties of these neurons. Hence, this algorithm may offer a special added value to world-wide leading brain research projects.

### Light-Sheet imaging

Large scale imaging of cleared tissues with a Light-Sheet microscope is a rapidly expanding field^13,15,17,42^. The shapes, dimensions, and locations of cortical barrel fields are critical for studies in the mouse somatosensory cortex, as well as for neurodevelopmental studies. For example, the barrels are notably altered following sensory deprivation during adolescence^36^, but the scope and significance of these changes in individual barrels remain largely unknown. Appling Intensify3D on LS data obtained from cleared adult mouse brains dramatically improved the detection and visualization of the barrel fields, indicating its applicability for such studies. At the microscale, we demonstrated that post-normalized scans of detected CChIs somata represent their real-life density, distribution, and composition compared to original scans, highlighting the importance of image normalization. Finally, to test the applicability of Intensify3D to diverse tissues we selected the mammary gland and heart, both of which present considerable challenges. We showed that with normalization we could extract the morphology of the milk ducts and buds by “simple” auto fluorescence. The heart is a complex organ composed of spaces and cavities that challenge imaging with a LS microscope. While this tissue challenged our tissue detections algorithm, the heart post-normalization showed superior uniformity, further strengthening the claim that images post-normalization represent the “real situation” better than uncorrected images. This predicts future use of the Intensify3D algorithm also for comparative studies that pursue dynamic changes in micro- and macrostructures, both in the brain and in other organs.

### Artificial data

Finally, to test the effects of normalization in a well-controlled milieu, we created an artificial data set of spheres in 3D and applied 4 types of distortions. Intensify3D managed not only to correct all of these distortions without adding any visible artifacts, but was also able to restore the data to the original intensity histogram in all cases (Fig. 5b). Moreover, correction empowered 3D object detection (Fig. 5c) and restored the basic statistics of the detected spheres (Fig. 5d). These results indicate that Intensify3D managed to correct both linear and logarithmic gradients across all 3 dimensions combined, and achieved it while preserving signal-to-noise ratios.

### General considerations

Notably, the definition of a “signal” primarily depends on the research question, and is subjective. Hence, applying a different size of the spatial filter (SFS), or selecting different maximum background intensity (MBI) levels will illuminate different structures in the resultant image; setting MBI too low will result in background regions of the image that will remain uncorrected, whereas combination of a high MBI with a small SFS will likely “correct” signal pixels and result in loss of information. In this context, any normalization process, if done carefully, can reduce signal variability. However, if the normalizing parameter (e.g., “housekeeping” gene, total protein concentration, RNA-seq or image background) is selected based on erroneous predictions, the correction process itself might introduce artifacts and mask information. For example, in cases where significant regions of the image are occupied by signal pixels, the normalization process would be compromised since the background in these regions will not be assessed correctly. For Intensify3D, errors might also occur if the background of the image is intrinsically different in intensity in one region of the stack as compared to another; for example, between different tissue types. Thus, making the basic decisions and defining the intrinsic assumptions of this tool is critical for achieving accurate normalization of microscopy signals based on background features. Another limiting factor comes from the attempt to “clean out” the signal from the image. Adding a machine learning approach may provide a more sophisticated way to improve finding of the signal-carrying pixels over the current selection, which is based on the definition of basic threshold and size filters. Lastly, the issue of normalization between images (along the Z axis) is not trivial. Because of the intrinsic differences in resolution between XY and Z in both SPIM^12^ and 2P^11^ point spread functions and the fact that Z step size is arbitrary, we treat each image as a separate data sample. To best match these data samples, we offer 3 types of between-image normalization: (1) The option of upper quantile normalization multiplies all of the pixels in an individual image by a constant (different for each image) so that the MBI value will match across the entire stack. This option will simply shift the intensity histogram but will not correct for any differences in the background histogram distribution (Fig. S3a). (2) Contrast stretch normalization linearly transforms each image in the stack so that the lower quantile (10th) and upper quantile values will match for the entire stack. This normalization will correct for differences in the “spread” of the intensity histogram (Fig. S3b). (3) Intensify3D records 10,000 quantiles from each image to precisely account for the intensity histogram of the background and signal which often occupies a very small number of pixels (<1%). Semi-quantile normalization will fit all the image quantiles lower than the upper quantile to match across the stack. From the upper quantile and above, the pixels will undergo the contrast stretch correction, assuming that these are the main fractions of pixels belonging to the signal. This normalization assumes that the background “behaves” similarly throughout the stack and that the differences observed should be corrected (Fig. S3c). Finally, there is the option not to correct across the depth of the stack but only by XY dimensions.

## Conclusions

Our current findings and analyses demonstrate that the Intensify3D tool may serve as a user-guided resource, correct sample- and technology-driven variations, improve the reproducibility, and add extractable information to numerous imaging studies in neuroscience research as well as in life sciences at large.

Given these advantages, we hope that our work will open an active discussion on matters of image normalization. We believe that image normalization has an integral role in any imaging experiment where numerical data is extracted. As in other fields of life sciences, normalization reduces variability between samples even when the experimental conditions are superb. Finally, Intensify3D might further be of value to time lapse fluorescence imaging platforms such as time lapse structural imaging^43,44^ or calcium imaging^45,46^ in which the fluorescence of the imaged sample is often compromised during imaging.

## Materials and Methods

### Mice

Two months old B57/B6 progeny mice derived from a cross of as loxp-stop-loxp-tdTomato (Ai14 - Stock No. 007914, Jackson Laboratories) with ChAT-IRES-Cre mice (Stock No. 018957, Jackson Laboratories) were employed.

### Ethics statement

All experiments were approved by the institutional animal care and use committees of The Hebrew University of Jerusalem (NS-15-14344-1, NS-13-13578- 4) which follow the National Research Council (US) Guide for Care and Use of 16 Laboratory Animals. All experimental protocols were approved by the University Ethics Committee for Maintenance and Experimentation on Laboratory Animals, The Hebrew University, Jerusalem, Israel.

### Microscopy

2 photon microscope: A Custom built 2 Photon Microscope, with excitation of 1050 nm and a 25x lens was used for *in vivo* imaging of CChIs. Imaging was driven by MScan software (Sutter Instruments, CA). Stacks of full-frame images (512 × 512 pixels) were acquired in Z steps of 1 µm. Each stack frame was an average of 5 images. CChIs Image stack is 271 µm in total depth (~30 µm from surface to 300 µm).

A La-vision Light-Sheet microscope ultra-microscope II (LaVisionBioTec) operated by the ImspectorPro software (LaVision BioTec) with Zoom body (Olympus) 0.63–6.3× lens situated with a Dipping cup LV OM DCC20 Dipping Cap [5.7 mm] including correction optics. Images were acquired by an Andor Neo sCMOS camera (2,560 × 2,160, pixel size 6.5 µm × 6.5 µm, Andor) in 16bit.

Cleared brains and tissues: Samples were attached with epoxy glue to the sample holder and placed in an imaging chamber made of 100% quartz (LaVision BioTec). The light sheet was generated by a Superk Super-continuum white light laser (emission 460  nm–800 nm, 1 mW/nm – 3 (NKT photonics)). Barrel cortex imaging was done at 2× magnification, 10 µm step size and blue excitation filter (peak – 470 nm/width − 40 nm) and a green emission filter (525/50). Mammary gland imaging was done with 2x magnification 10 µm step size (150 images), blue excitation filter (peak – 470 nm/width −40 nm) and a green emission filter (525/50). Heart tissue was imaged in 0.8x with 5 µm step size (800 images). For CChIs, imaging was done at 5x magnification, 1 µm step size (later down sampled to 4 µm per image with Image J size adjust interpolation) and a far-red excitation (640/30) and emission filter (690/50).

### Procedures

#### *In vivo* 2-Photon

For the *in vivo* 2-Photon experiments, we administered mice with Rymadil analgesia (200 mg/kg body weight, 200 µl injection volume). Anaesthetized mice were put in a stereotactic frame (Narishige, Japan) and a small craniotomy (3 mm in diameter) was made over the right barrel cortex (2 mm caudal, 3 mm lateral to Bregma); dura was not removed. A 3 mm glass window was implanted over the craniotomy and sealed with VetBond (3M). CChIs were imaged through the cranial window. ChAT-IRES-CreXAi14 mice were anaesthetized with isoflurane (1–2% by volume in O2 LEI medical). Anaesthetized mice were euthanized by cervical dislocation.

#### iDISCO clearing and staining

For the iDISCO-cleared brain experiments as well as mammary and heart, ChAT-IRES-CreXAi14 mice were anaesthetized with isoflurane (1–2% by volume in O2 LEI medical), administered with an intra-peritoneal injection of 200 mg/kg sodium pentobarbital. Following trans-cardial perfusion with 1xPBS solution and then 10% Formaldehyde in 1xPBS solution, the mouse brains, mammary gland, and heart were collected and used for iDISCO clearing as described^13^. For staining of the tdTomato expressing cells we used an anti-RFP antibody (Rockland, 600-401-379) followed by Alexa-647 conjugated Donkey anti-Rabbit secondary antibody (Jaxson immunoResearch, 711-605-152), following manufacturer’s instructions.

#### Software

Normalization tool and graphical user interface were designed with MATLAB (Simulink). 3D image rendering was done using the FIJI (ImageJ), 3D viewer plugin. Neuronal reconstruction was performed in Vaa3D (Allen Institute) by N.V. in a “blind manner”. Neuronal diameter analysis was done with NEURON (Yale). External MATLAB and ImageJ scripts that were used in the algorithm are detailed under Supplementary Table 1. Detailed instructions, source code and standalone files are accessible at GitHub repository - https://github.com/nadavyayon/Intensify3D and in Supplementary Fig. S1. Example data sets and movies are also available via Google Drive link published in the GitHub repository as well.

#### System requirements

The normalization algorithm could potentially run on any operating system, since the use of memory or CPU power mainly depends on the size of the images and on parallel processing. In the user GUI, one can select the number of cores to use. Using more cores may enable simultaneous processing of more images, which will be faster but requires more memory. Users who do not possess an active MATLAB License can use the standalone version which requires an additional download of Free MATAB library files (500–700 MB depending on operating system). To conserve RAM memory, the basic statistics of each image (quantiles or mean intensity) are logged and then used for quantile or mean-based normalization across the image stack. Normalized images are then saved in a separate folder as an image series. To cope with large data sets, the algorithm takes advantage of MATLAB parallel processing (controlled by the user) and simultaneously corrects multiple images in the stack given that the RAM memory is sufficient. For memory conservation purposes, the estimated tissue region is saved in a form of a support image in a distinct folder. Using a standard PC with a core i7-4930 K and 64 gb of RAM −7 images of size 512 × 512 may be corrected per 1s. A typical Light-Sheet image also requires background estimation, and will take 5s per image. Naturally, this time estimation depends on the number/speed of processors and available RAM that the PC has.

## Electronic supplementary material

Supplementary information

Movie 1

Movie 2

Movie 3

GUI figure

source code for Intensify3D

Graphical user interface for Intensify3D
