# Supplementary material for: Intensify3D: Normalizing signal intensity in large heterogenic image stacks
Source: Sci Rep. 2018 Mar 9;8:4311. doi: 10.1038/s41598-018-22489-1 (PMC5844907; doi:10.1038/s41598-018-22489-1)
Supplement: Supplementary file 1 — Supplementary information [file 41598_2018_22489_MOESM1_ESM.pdf]

Intensify3D: Normalizing signal intensity in large heterogenic image stacks

Nadav Yayon[1,2], Amir Dudai[2,3], Nora Vrieler[2,3], Oren Amsalem[2,3], Michael London[2,3] and Hermona Soreq[1,2,*]

*To whom correspondence should be addressed: Prof. Hermona Soreq, Department of Biological Chemistry, The Life Sciences Institute and The Edmond and Lily Safra Center for Brain Sciences, The Hebrew University of Jerusalem, Jerusalem, Israel 9190401. hermona.soreq@mail.huji.ac.il.

[1]Department of Biological Chemistry, The Life Sciences Institute, The Hebrew University of Jerusalem, Jerusalem, Israel [2]The Edmond and Lily Safra Center for Brain Sciences (ELSC), The Hebrew University of Jerusalem, Jerusalem, Israel [3]Department of Neurobiology, The Life Sciences Institute, The Hebrew University of Jerusalem, Jerusalem, Israel 9190401

Supplementary Figures

Supplementary Figure S1

a

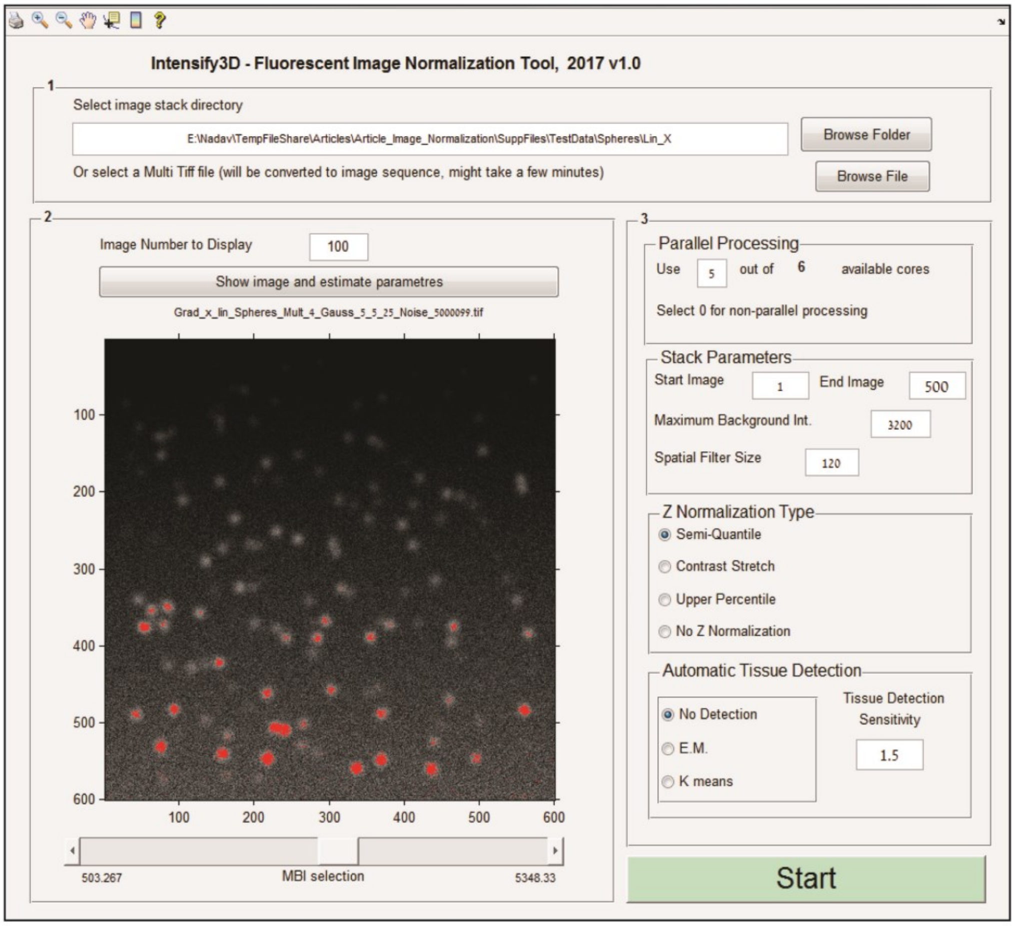

b

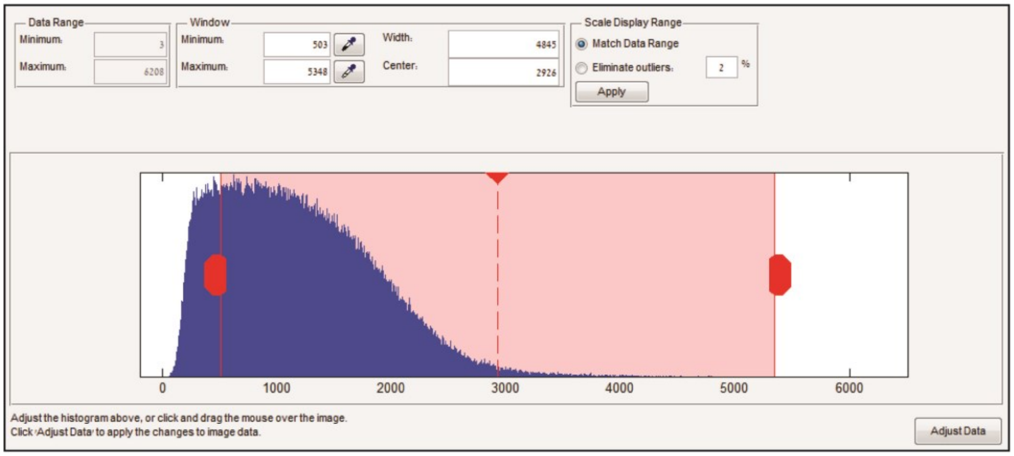

Supplementary Figure S2

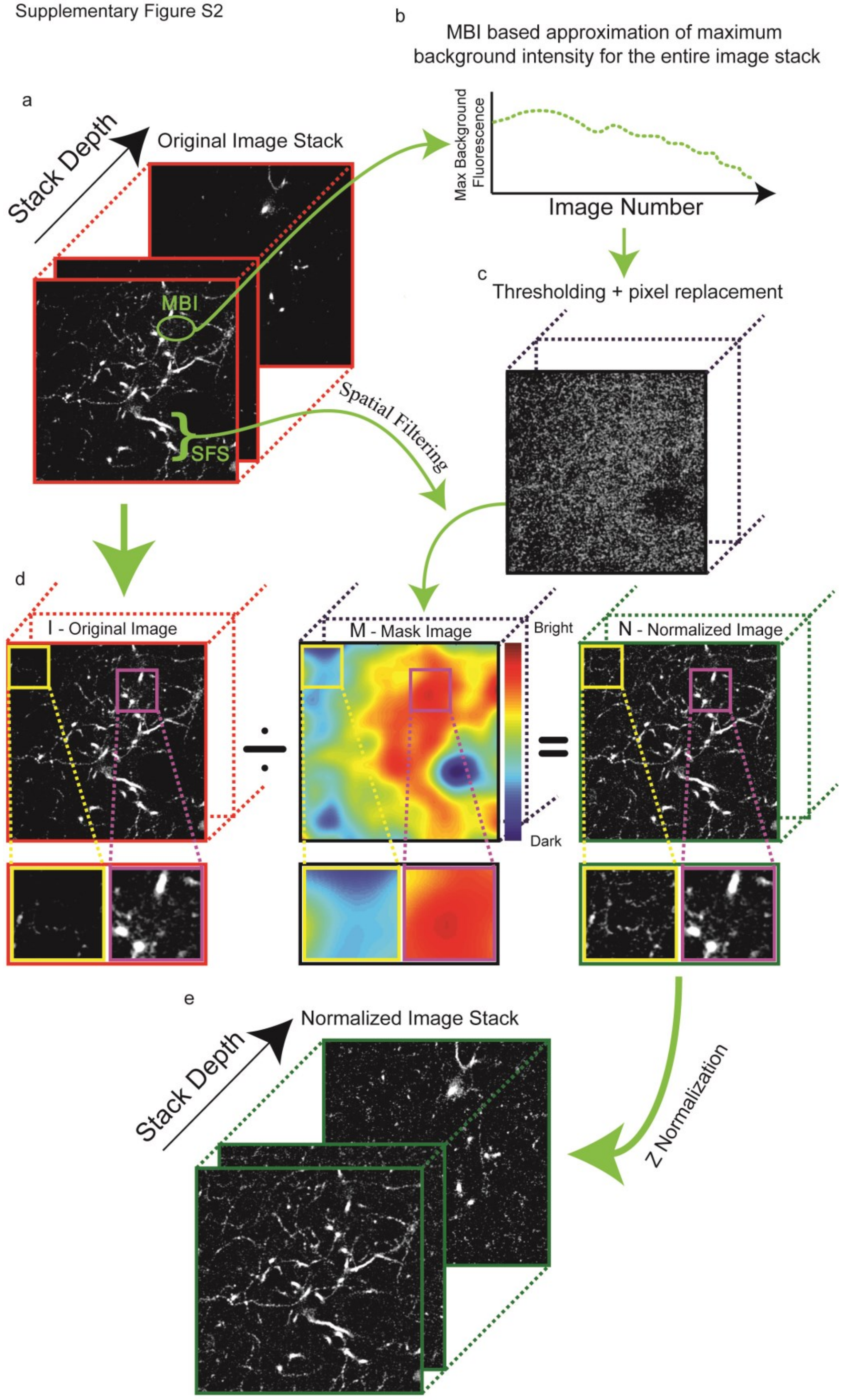

Supplementary Figure S3

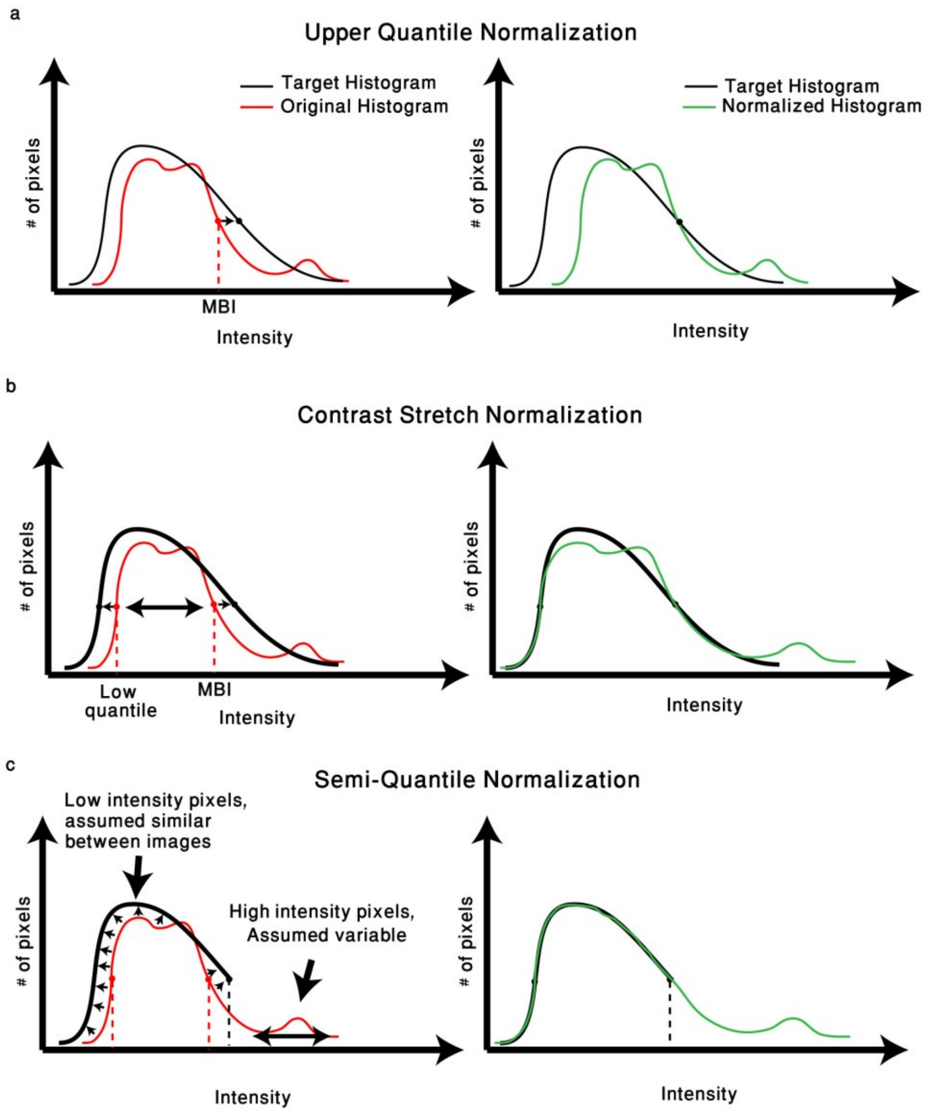

Supplementary Figure S4

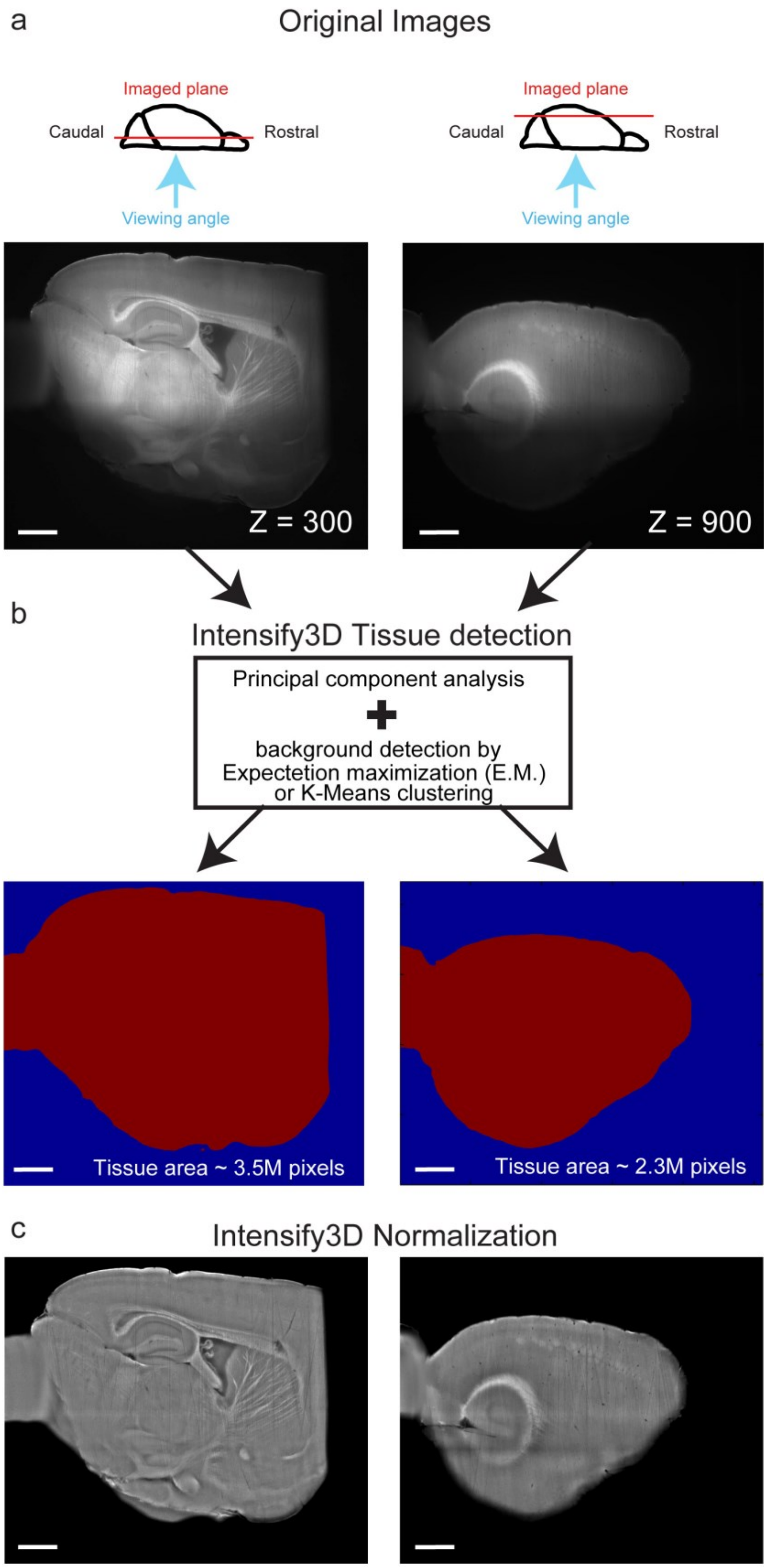

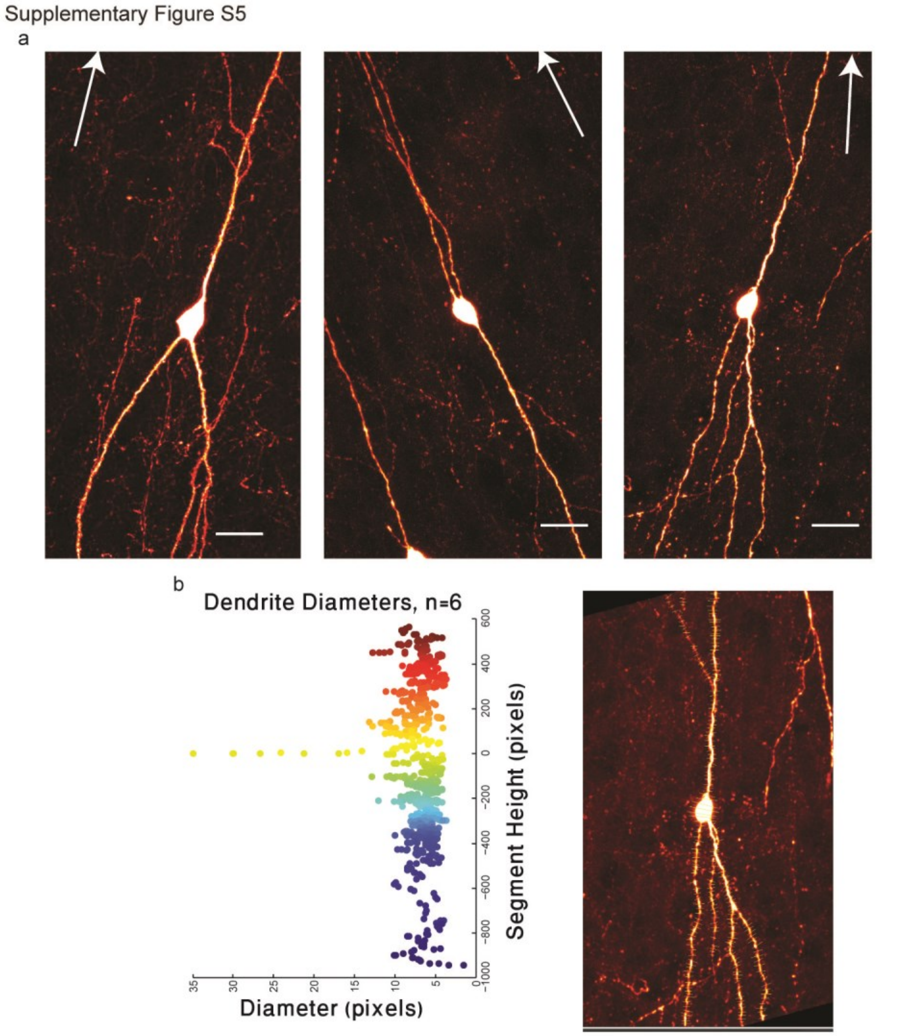

Figure S1. Graphical user interface (GUI) Manual.

Before starting please read the manuscript and make sure the assumptions of normalization are met. Intensify3D can correct an unlimited number of images since it operates in a serial manner. Hence, it only supports image sequences. However if your image stack is in multi-Tiff format there is an option in the GUI to convert the file to individual images. The *.tif files should ideally be unprocessed data in a 12 or 16bit format. Memory requirements depend on image size and parallel processing. Based on our experience, the maximum requirements are 750 bytes/pixel. Thus, processing a single Light-Sheet image of 2560x2160 pixels will require ~ 4Gb of RAM from each processor + 4Gb for general processes. For example, if your PC has 4 cores, it is

possible to analyze 4 Light Sheet images simultaneously, which will require 20Gb of RAM. **It is highly recommended to start with a few representative images (~20), adjust the parameters and only then run the process on the entire stack.**

<u>Operation instructions and GUI options:</u>

a. The graphical user interface is divided to 3 panels:

Panel 1 – Folder or file selection: Here the user selects the directory containing the individual images in the stack in TIFF format. Alternatively, if the images are in multi-TIFF format, the user should select the "browse file" option and the multi-Tiff will be converted to multiple file form in a directory carrying the file name.

Panel 2 – Estimate your background: The objective of this section is to assist the user to select the ideal maximum background intensity (MBI) in a single image. This value will be used by Intensify3D to estimate the background across all images in the stack. "Image number to display" is used to select a representative image from the stack that carries a clear signal. Once the image has been selected, pressing the "show image and estimate parameters" button displays the requested image is displayed, a brightness contrast adjustment window opens (**b**) and an initial estimation of the MBI is assigned based on the 99th percentile of intensity potentially showing only signal pixels in red. Next, the user should adjust the MBI selection with the dedicated slide bar at the bottom of the image in the following order: (1) adjust brightness and contrast (2) move slide bar to set MBI (performing 2 before 1 will show a distorted selection of the MBI). Repeat 1->2 until satisfied with the result*. The matched value for the MBI will be set in the "stack parameters" section in panel 3.

Panel 3 – Setting run parameters: The parallel processing section is very useful when analyzing large image stacks. The GUI detects how many cores your CPU has and offers the user the option of how many of them will be dedicated to the run. If your MATLAB license does not include the

parallelization package, select 0 and work without it (this limitation does not apply to the standalone version). The “stack parameters” section defines the first and last image that would be processed in the stack, the MBI (described above) and the spatial filter size. Spatial filter size determines the frequency of intensity changes that would be corrected by Intensify3D. The minimum value for this parameter should be at least twice the diameter of the largest signal structure. Lower values could affect the signal. “Z normalization type” section allows selection of the desired normalization type across the images in the stack (for more information see main text and supplementary figure S3). Last, Intensify3D has the ability to detect the background or tissue area in an image based on 2 clustering algorithms: K means and Expectation Maximization (E.M.). This option should be explored for images where not all the image area is relevant for normalization and is critical in such images. The sensitivity of the tissue detection should be experimented by the user to fit to the specific image set (for more information see main text and supplementary figure S4).

Last, after running Intensify3D, The “Start” will change to messages regarding the run's progression.

*intuition for selection of the MBI value: The correct approximation of the image background depends on “cleaning out” the signal pixels by thresholding and spatial filtering. High brightness signal pixels can affect the ability of the spatial filter. **The MBI should be set so that most signal pixels will be removed without removing background pixels**. Notice the red-labeled pixels in the example image of bright spheres. Lowering the MBI would result in removing background pixels and increasing the MBI would retain more signal pixels. Both ways will lead to a sub-optimal estimation of the background.

Figure S2. The basic normalization process of a 2-photon image stack with Intensify3D.

a. For a given image stack (red frames) the user is encouraged (but not obliged) to select 2 parameters: Maximum Background Intensity (MBI, green circle) and Spatial Filter Size (SFS,

green parenthesis), both derived from a single image in the stack. **b.** The MBI is matched with the image quantile and that quantile is then measured in the entire image stack to serve as an image specific MBI estimate. **c.** Initially the pixels in the image that are brighter than the MBI are replaced by values derived from an empirical distribution based on the rest of the pixels in the original image*. Next, the image is filtered based on the SFS with a Savitzky-Golay low pass filter to generate mask image M that should now depict the intensity gradients in the background. The mask image is standardized (max value = 1). **d.** Normalization by division of the original image I (left) by the mask image M (middle) produces image N (green frame). Note the magenta rectangles, highlighting a relatively unchanged region (with high background intensity), and the yellow rectangles, representing a region that was intensified post-normalization. Between-image normalization (Z axis normalization) is achieved either by upper quantile, contrast stretch or semi-quantile normalization (see figure S5 and methodological outline for details).

*image presented in enhanced brightness

Figure S3. Z normalization types and the expected effect on image data. Each normalization approach is presented relative to the pixel histogram of the "ideal case" imaging setup (Target histogram). Left/right panels are before/after normalization **a.** Upper quantile normalization will multiply each image in the stack by a different constant to match the upper quantile (extrapolated from the maximum background intensity) value for the entire stack. **b.** Contrast stretch normalization linearly transforms each image in the stack so that the lower percentile (10th) and Upper quantile values will match for the entire stack. **c.** Semi-quantile normalization will force 10000 image quantiles lower than the upper quantile to match across the stack. From the upper quantile and above, the pixels will undergo the contrast stretch correction.

Figure S4. **Automated tissue detection**. **a.** Representative pre- normalized Light-Sheet scans of cleared adult mouse brains from 2 district regions: Z = 300, mid sagittal and Z = 900, lateral**.** Top

illustrations present the imaged planes and the orthogonal viewing angle of the Light-Sheet microscope. **b.** The value of each pixel and of neighboring pixels serve as a basis for PCA analysis and dimensionality reduction. An iterated expectation-maximization (E.M.) algorithm is used to cluster pixels that belong to the image's background **(blue)** and the tissue **(red). c.** The image is normalized only according to tissue pixels, ignoring the background and thus allowing accurate normalization even if the tissue area changes significantly throughout the image stack. Scale bar represents 1mm.

Figure S5. Cortical Cholinergic Interneurons (CChIs) with representative bipolar morphology. a. Coronal cortical tissue sections (30μm thick) were extracted from a 1 month old ChAT-IRES-Cre X tdTomato-loxp (Ai14) mouse and imaged with a confocal microscope (fv-10i, Olympus) at 60X magnification, 1μm Z-step. Images are projected along the Z axis. Scale bar is 20μm. White arrows indicate the orientation to the surface of the cortex. **b.** Quantification of dendrite and soma diameters across cortical depth. **Right panel**. Multiple line ROIs (yellow thin lines) were manually selected along the neuronal dendrites and soma (n = 6 neurons). Images were rotated so that the cortical surface will point up. The dendrite diameter was estimated from the maximum half-width of the intensity profile along the line. **Left panel.** data from all neurons was centered (soma is 0), merged and smoothed with a moving average (5 lag/lead). All units are in pixels of original images. Color code matches the one in Figure 2 d.

Supplementary Movies

Movie 1. 3D rendering of 2-Photon image stack. A movie showing a 360° rotation of a 3D rendering (Fiji, 3D viewer plugin) based on original images (left) and corrected images (right). Last 10s show the same rendering but with 50% transparency. The image stacks are shown at the same contrast levels.

Movie 2. Light-Sheet auto fluorescence sweep through pre- and post-normalization. Light-Sheet scan in the blue/green excitation emission spectrum of cleared mouse hemisphere samples before (**left)** and after (**right) Intensify3D correction.** Last 10s show the same images but with a higher intensity threshold. The images are shown at the same contrast levels.

Movie 3. 3D rendering of barrel fields based on auto-fluorescence iDISCO scans before (left) and after (right) Intensify3D normalization.

__Supplementary files__

1) "2Photon_CChIs_Before_and_After.mp4"- Movie presenting 3D rendering of 2-photon imaged CChIs before and after normalization.
2) "barrel_Before_After.mp4": Movie of identified barrel fields from light-sheet imaging before and after normalization.
3) "LightSheet_Before_After_Thr.mp4": Movie of Light Sheet images while scanning along the Z axis before and after normalization, side by side.
4) Code – MATLAB code and standalone installation files with instructions.

Supplementary Table 1 – Open source MATLAB and Image j scripts used in the normalization algorithm.

Platform	Function name	Use	author
MATLAB	mixGaussEm	Gaussian mixture model	Mo Chen (sth4nth@gmail.com)
MATLAB	neighbourND	Get indices of neighboring pixels	Ronald Ouwerkerk

MATLAB	Savitzky_Golay	Savitzky Golay filtering	By Image Analyst
MATLAB	parfor_progress	Track progression of a parallel process	Jeremy Scheff<br>http://www.jeremyscheff.com/
MATLAB	HartigansDipSignifTest	calculates Hartigan's DIP statistic and its significance for the empirical p.d.f XPDF	F. Mechler (27 August 2002)
MATLAB	emprand	Generates random numbers from empirical distribution of data	Durga Lal Shrestha<br>eMail: durgals@hotmail.com<br>% Website:<br>http://www.hi.ihe.nl/durgalal/index.htm
Image j	3D Viewer	Visualization and rendering of 3D images	Benjamin Schmid, Albert Cardona, Mark Longair, Johannes Schindelin.<br>http://3dviewer.neurofly.de
Image j	3D object counter	counts the number of 3D objects in a stack	Fabrice P. Cordelieres and Daniel James White.<br>http://imagejdocu.tudor.lu/doku.php?id=plugin:analysis:3d_object_counter:start
